# CircMIB1 inhibits glioma development and progression through a competing endogenous RNA interaction network

**DOI:** 10.3389/fmolb.2024.1513919

**Published:** 2024-12-04

**Authors:** Simin Chen, Longping Li, Wei Xu, Nanjiao Xie, Huiting Xu, Yongjun Zhou, Ying Zou, Kai Yi, Zuping Zhang

**Affiliations:** ^1^ Department of Clinical Laboratory, Yiyang Central Hospital, Yiyang, Hunan, China; ^2^ School of Xiangya Basic Medical Science, Central South University, Changsha, Hunan, China

**Keywords:** CircMIB1, glioma, MiR-1290, ceRNA, prognostic, biomarker

## Abstract

**Introduction:**

The critical role of circular RNAs as non-coding RNAs in glioma has been extensively investigated. Therefore, we aimed to explore the role and potential molecular mechanisms of circRNA-mind bomb homolog 1 (circMIB1) in gliomas.

**Methods:**

RNA sequencing was used to analyze the expression profiles of circRNAs in glioma tissues and normal brain tissues. Quantitative real-time polymerase chain reaction was implemented to examine the levels of circMIB1 in glioma cells and tissues. The circMIB1 was identified as a cyclic RNA molecule by DNA nucleic acid electrophoresis and ribonuclease R assay. The relationship between circMIB1 expression and the prognosis of glioma patients and its potential as a biomarker were analysed using Kaplan-Meier, Receiver operating characteristic curves, and Principal component analysis. Bioinformatics analysis predicted the miRNAs that bind to circMIB1 and their downstream targets, and analysed the functions of these genes.

**Results:**

Firstly, a novel circRNA molecule termed circMIB1 was identified and validated by RNA sequencing. The expression of circMIB1 was significantly downregulated in glioma cells and tissues, and was closely associated with the tumor grade and survival prognosis of patients with glioma. Hence, it may be useful as a biomarker for glioma. Secondly, it was predicted that circMIB1 binds to hsa-miR-1290 based on bioinformatics analysis, which was significantly upregulated in glioma cells and tissues, and correlated with the tumor grade and overall survival of patients. Thirdly, through a series of bioinformatics analyses identified six genes downstream of hsa-miR-1290 that were significantly associated with glioma expression and prognosis, these genes are associated with cell cycle, cell necrosis and cell circadian rhythms.

**Discussion:**

CircMIB1 may play a role in inhibiting glioma development through the hsa-miR-1290 competitive endogenous RNA interaction network, these findings provide new ideas and directions for the diagnosis and treatment of glioma.

## Introduction

Gliomas are among the most common primary brain tumors, accounting for approximately 30% of all brain and central nervous system malignancies. Gliomas are highly aggressive and are linked to a high mortality rate, thus, these tumors have become a serious threat to human health (Chew et al., 2018). Although some progress has been made in the treatment of this disease, there is currently a lack of effective and noninvasive diagnostic methods (Cheng et al., 2020; Stupp et al., 2005). Consequently, gliomas represent a major source of both health-related challenges and economic burden due to their aggressive nature and the costs of ongoing treatment (Hervey-Jumper and Berger, 2014). In addition, complete removal of high-grade glioma (HGG) through surgery is typically difficult. Moreover, glioma stem cells exhibit enhanced self-renewal, division, and proliferation, and are often resistant to conventional therapies, such as chemotherapy and radiotherapy, contributing to tumor recurrence and poor prognosis (Ostrom et al., 2015). Therefore, elucidating the molecular regulatory mechanisms during glioma development and identifying molecular markers can further advance the genetic diagnosis of clinical glioma and improve therapeutic efficacy.

Circular RNA (circRNA) is a new type of non-coding RNA with a covalently closed circular structure (Acunzo et al., 2015). CircRNAs are resistant to ribonuclease R (RNase R) degradation due to their covalently closed circular structure, which enhances their stability in biological fluids such as blood compared to linear RNAs. Therefore, circRNAs have great application value in the field of noninvasive diagnostics, and have emerged as a new class of potential tumor markers (Zhang et al., 2016). Tang et al. found that gastric cancer tissue-derived circKIAA1244 could be used as a new circulating biomarker for gastric cancer (Tang et al., 2018). Moreover, Li et al. demonstrated that hsa_circ_002059 may be a novel and stable biomarker for the diagnosis of gastric cancer from patient tissue and plasma samples (Li et al., 2015). In recent years, numerous studies reported that the expression of circRNAs in gliomas differs significantly. The expression of certain circRNAs varies depending on the degree of malignancy (Lu et al., 2019), and these circRNAs play a crucial role in glioma development by regulating both oncogenes and tumor suppressor genes. This process affects the invasion and metastasis of tumors, as well as the staging and grading of tumors (Sun et al., 2020). Studies have shown that some circRNAs correlate with the pathological grading of glioma, as well as the survival rate of patients with glioma. For example, circCDK14 expression correlates with the World Health Organization pathological grading of glioma (Chen et al., 2022), and hsa_circ_0001649 may be an independent prognostic indicator for patients with glioma after surgery (Wang et al., 2018). In addition, some circRNAs in gliomas act as miRNA sponges, sequestering the activity of miRNAs and indirectly regulating the expression of target genes (Lei et al., 2019; Shi et al., 2019), others behave as RNA-binding proteins and regulate protein interactions (Lou et al., 2020; Du et al., 2016), and still others have been reported to be translatable (Yang et al., 2018; Zhang et al., 2018). Currently, the exact functions and molecular mechanisms of most circRNAs in glioma are unknown. There is also a need to determine the expression and mechanism of action of circRNAs involved in the pathogenesis of glioma. Further studies are warranted to confirm whether circRNAs can be used as therapeutic targets or diagnostic and prognostic markers for glioma.

The circRNA-miRNA-mRNA pathway plays an important role in the development and progression of glioma, though the full extent of its involvement and mechanisms remains under investigation. Most circRNAs can be used as sponges of miRNA to regulate gene expression; this is key to clarifying the molecular mechanism of circRNA (Zhong et al., 2018). Studies have shown that circHIPK3 can also promote glioma progression by acting on its downstream target miR-654 and further regulating the circHIPK3/miR-654/IGF2BP3 network pathway (Jin et al., 2018). Another study also showed that deletion of circ_0074026 promotes apoptosis by regulating miR-1304 to block cell growth, migration, and invasion, providing a new therapeutic target for patients with gliomas (Chen et al., 2020). However, there may be other circRNAs regulated by the competing endogenous RNA (ceRNA) network in gliomagenesis and progression that have not yet been identified.

This study was performed to further investigate the influence of differentially expressed circRNAs on the occurrence, development, and prognosis of glioma. Firstly, the differential expression of circRNAs in glioma tissues and normal brain tissues was screened using circRNA high-throughput sequencing technology. Secondly, the study of unreported low-expression circRNAs in gliomas (circMIB1) was planned. Thirdly, we sought to examine their correlation with clinicopathological features of patients with glioma and reveal the mechanism by which they inhibit gliomagenesis through the ceRNA regulatory network. The objective was to provide new directions and ideas for the diagnosis, prognosis prediction, and molecular-targeted therapy of glioma.

## Materials and methods

### Tissues specimens and cell lines

In this study, 76 primary glioma samples were collected from glioma patients, and 17 nontumor brain tissues were obtained from patients with brain trauma or epilepsy. These samples were obtained from the Department of Neurosurgery of Yiyang Central Hospital (Yiyang, China). Following surgical resection, all specimens were frozen in liquid nitrogen and stored at −80°C. All participants or their guardians provided written informed consent for their participation. This study was approved by the Ethics Committee of Yiyang Central Hospital (Ethics approval reference number: V2.02024JJ9575).

The normal human glial cell line HEB was acquired from the Cell Bank of the Type Culture Collection of the Chinese Academy of Sciences (Shanghai, China). Human glioma cell lines SF126 (a glioblastoma cell line), U251, and U87 (glioma cell lines) were obtained from the American Type Culture Collection (ATCC, Manassas, VA, USA). The cell lines were grown in Dulbecco’s modified Eagle’s medium supplemented with 10% fetal bovine serum (Gibco, USA) at 37°C and 5% CO_2_.

### RNA sequencing and bioinformatic analyses

Five glioma samples and four non-tumor brain tissues were processed and RNA sequenced by Lc-Bio Technologies (Hangzhou, China). A larger sample size would be ideal to ensure statistical significance and biological reproducibility in future studies. Results were screened for significant differences in the expression of circRNAs using the edgeR software package (https://www.Bioconductor.org/) to identify differences with |log_2_fold-change (log_2_FC)| ≥1 and *p*-values < 0.05. The results showed significant differences in circRNA expression levels between glioblastoma (GBM) and non-tumor brain tissues.

### Quantitative real-time polymerase chain reaction (qRT-PCR)

Total RNA isolation was performed using TRIzol reagent (Takara, Japan) according to the instructions provided by the manufacturer. Individual RNA samples (1 μg per sample) were converted to complementary DNA (cDNA) using the Prime Script RT kit (Takara) and the cDNA conversion step used a DNase (Takara) treatment to eliminate residual genomic DNA, and a RT-free control to check for genomic DNA contamination was used. While cDNA detected on an ABI Prism 7,500 Sequence Detection System (Applied Biosystems, USA) using the Hieff^®^ qPCR SYBR Green Master Mix (Yeasen, China). Of note, glyceraldehyde-3-phosphate dehydrogenase (GAPDH) was used as an endogenous control. The qRT-PCR was performed using primers obtained from Bioengineering Biologicals (Changsha, China). The relative quantitative expression levels of circRNAs, miRNAs, and mRNAs were compared with those of the endogenous reference and analyzed using the 2^−ΔΔCT^ method. Divergent primers were used to detect post-splicing junctions of circRNAs, and convergent primers were used to detect linear mRNAs. The primer sequences used are described in Supplementary Table S1.

### Nucleic acid electrophoresis

The cDNA and genomic DNA amplification products were separated by 2% agarose gel electrophoresis in tris-acetic acid-ethylenediaminetetraacetic acid buffer, the DNA was separated by electrophoresis at 120 V for 25 min, and the DNA sizes were compared with those of Marker L (50–5,000 bp) (Sangon Biotech, China). Ultraviolet light was used to detect the bands.

### RNase R assay

Original RNA (2 μg) was added to 2U RNase R (GeneSeed, Guangzhou, China), and a large amount of linear RNA could be digested by incubation at 37°C for 30 min. The reverse-transcription reaction was carried out by inactivating RNase R at 70°C for 10 min. The other untreated group was tested for circMIB1 and MIB1 levels by qRT-PCR using an internal control (i.e., GAPDH) as the calculation standard.

### miRNA binding site prediction

The miRNA bindig sites were predicted using Circular RNA Interactome (https://circinteractome.nia.nih.gov/), and data from the GEO database (GSE90603, https://www.ncbi.nlm.nih.gov/gds/). The screening criteria were |log2FC|≥1 and *p*-values < 0.05, totaling yielding 59 differential miRNAs.

### Bioinformatic analyses of mRNA

The Cancer Genome Atlas (TCGA, https://portal.gdc.ancer.gov/) includes a total of 393 cases of GBM and 508 cases of low-grade glioma (LGG). RNA expression data of normal brain tissue from the Genotype-Tissue Expression (GTEx) were downloaded from the UCSC Xena database (https://xenabrowser.net/datapages/). RNA transcriptomes of glioma specimens were obtained from The Cancer Genome Atlas (TCGA; https://portal.gdc.cancer.gov/). Subsequently, the RNA-sequencing data were log_2_ transformed and normalized for transcription per kilobase (TPM) values, and RNA-sequencing data (RSEM values) to make the gene expression profiles of the different platforms comparable. The R “limma” package was used to analyze the differential mRNA expression in the glioma tissues and normal brain tissues.

We also used GEPIA2 (Gene Expression Profiling Interactive Analysis, version 2) (http://gepia2.cancer-pku.cn/#index) to perform survival analysis and disease-free status analysis of differentially expressed genes in glioma. The Chinese Glioma Genome Atlas (CGGA, http://www.cgga.org.cn/) was also utilized for validation. Furthermore, we performed Gene Ontology (GO) enrichment analysis and pathway analysis. Finally, using the R package ggplot2 (https://www.r-proje ct.org/) in R version 4.2.2, dot plots were plotted to visualize valid terms and significant pathways for the GO enrichment analysis (*p* < 0.05).

### Statistical analysis

Data were analyzed using unpaired Student’s t-test or one-way analysis of variance (ANOVA) after confirming normal distribution of the data with the Shapiro-Wilk test, using GraphPad Prism eight software (GraphPad Software Inc, San Diego, CA, USA). All experiments were performed at least in triplicates. Pearson’s correlation coefficient was employed to examine correlations. Data are presented as the mean ± standard deviation. The *p*-values < 0.05 indicate statistically significant differences.

## Results

### High-throughput sequencing to screen differentially expressed circRNAs

**FIGURE 1:**
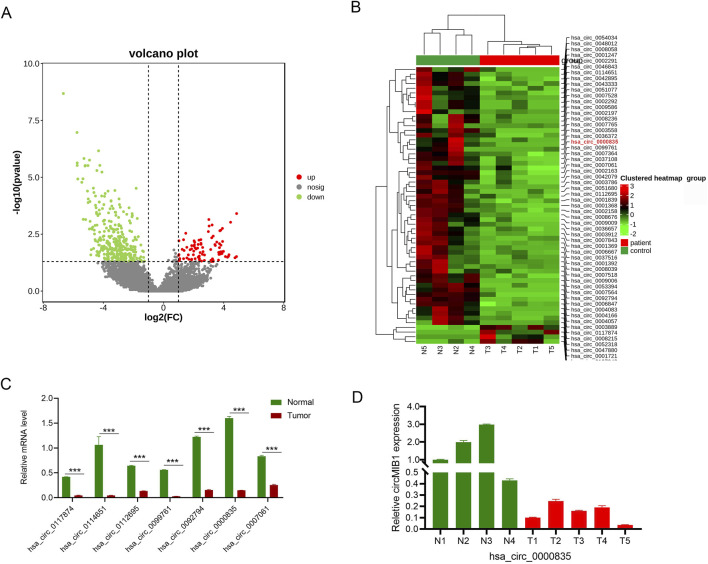
High-throughput sequencing to screen differentially expressed circRNAs. **(A)** Volcano plot of diferentially expressed circRNAs. The green dots represent downregulated circRNAs, whereas the red dots represent upregulated circRNAs in glioma compared with non-tumor tissue. **(B)** Clustered heatmap of circRNAs exhibiting significant differential expression in glioma tissues and non-tumor brain tissues (|log2 fold-change| ≥2, *p* < 0.05). Red and green indicate upregulated and downregulated circRNAs, respectively; hsa_circ_0000835 is labeled in red. **(C)** Downregulated circRNAs in glioma tissue. **(D)** Expression of circMIB1 in four and five samples of non-tumor and tumor tissues, respectively. Data are presented as the mean ± SD.*p < 0.05; **p < 0.01; ***p < 0.001; ****p < 0.0001.

We performed circRNA high-throughput sequencing on four normal brain tissues and five glioma tissues. A total of 1,248 differentially expressed circRNA molecules were identified, of which 158 were upregulated and 1,090 were downregulated in glioma. The 652 differentially expressed circRNA molecules were recognized based on set criteria (|log2FC| ≥2 and *p*-values < 0.05), including 67 genes with upregulated expression and 585 genes with downregulated expression (Figure 1A). Sixty differentially expressed circRNA molecules were subsequently selected for cluster analysis based on statistical significance (|log2FC| ≥2 and *p*-values < 0.01), there was an overall decrease in circRNA expression abundance in cancer tissues compared to normal tissues. Consequently, we focused on circRNA molecules with downregulated expression and identified a significantly downregulated circRNA molecule, namely, hsa_circ_0000835 (circMIB1) (Figure 1B). Seven circRNA molecules with downregulated expression were randomly selected for RT-PCR validation. The validation results were consistent with the sequencing results, thereby confirming the findings (Figure 1C). Among all the validated genes, circMIB1 demonstrated the most significant expression difference in four normal brain tissues and five glioma tissues, and it has not been studied thus far in glioma (Figure 1D). Therefore, it was selected as the final target circRNA for further analysis.

### Low expression of circMIB1 in glioma cells and tissues

**FIGURE 2:**
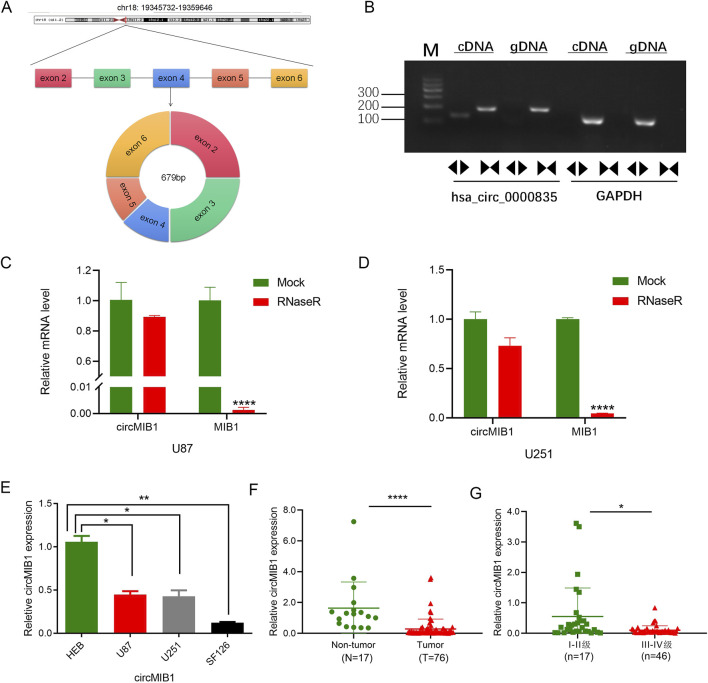
Low expression of circMIB1 in glioma cells and tissues. **(A)** Schematic showing the circMIB1 derived from exons 2–6 circularization of MIB1. **(B)** PCR was performed to analyze the circular RNA characterization of the circMIB1 (hsa_circ_0000835) using the divergent and convergent primers for amplification from the cDNA and genomic DNA (gDNA) of U251 cells, respectively. **(C, D)** RT-PCR analysis of circMIB1 and linear MIB1 mRNA, in presence or absence of RNaseR treatment for 30 min. **(E)** The expression level of circMIB1 in glioma cell lines (U87, U251 and SF126) and normal brain glial cells (HEB) was quantified by qRT-PCR. **(F, G)** CircMIB1 levels in 76 glioma and 17 non-tumor brain tissues, and in 30 patients with I-II grade glioma and 46 patients with III-IV grade glioma. Data are presented as mean ± SD.*p < 0.05; **p < 0.01; ***p < 0.001; ****p < 0.0001.

We identified circMIB1 (hsa_circ_0000835) through the circBase database as being formed by reverse splicing of its parental gene MIB1 localized in exons 2–6 on chromosome 18 at positions 19,345,732–19,359,646, with a length of 679 bp (Figure 2A). MIB1 is an E3 ubiquitin ligase, which is widely expressed in adult brains and plays an important role in the morphogenesis of neurons. It interacts with certain proteins to form a network hub (Yoon et al., 2012), which plays a broad and powerful control role in the development of cells, tissues and organs (Mertz et al., 2015). However, the function of circRNAs derived from MIB1 in glioma remains unknown. The divergent and convergent primers were designed based on the characteristics of circMIB1. Using PCR analysis, we found that the divergent primers successfully amplified circMIB1 from cDNA of U251 cells, whereas genomic DNA (gDNA) did not. In contrast, the linear transcript MIB1 was amplified with the convergent primers. Agarose gel electrophoresis was used to verify that circMIB1 is indeed a circRNA molecule (Figure 2B).

Following treatment of cDNA with ribonuclease R (RNase R), circMIB1 was found to be resistant to RNase R by qRT-PCR; however, its linear MIB1 was disrupted by RNase R in U87 and U251 cells, suggesting that circMIB1 is unaffected by RNase R and has a circular structure (Figures 2C,D). The expression of circMIB1 in normal brain astrocyte cell line HEB and malignant glioma cell lines U251, U87, and SF126 was evaluated. The expression of circMIB1 was significantly lower in malignant glioma cell lines *versus* normal brain astrocyte cells (Figure 2E). The downregulated expression of circMIB1 in glioma tissues was further investigated in a larger sample size using RT-PCR. The analysis showed that the expression levels of circMIB1 were significantly lower in 76 GBM tissues compared with 17 normal brain tissues. Among 76 glioma tissues of different grades, circMIB1 expression had significantly lower expression in high-grade tissues *versus* low-grade tissues (*p* < 0.05) (Figures 2F, G). These data indicated that circMIB1 has a circRNA profile and is lowly expressed in glioma.

### CircMIB1 expression correlates with patient clinical characteristics and haspotential as a diagnostic marker for gliomas

**FIGURE 3:**
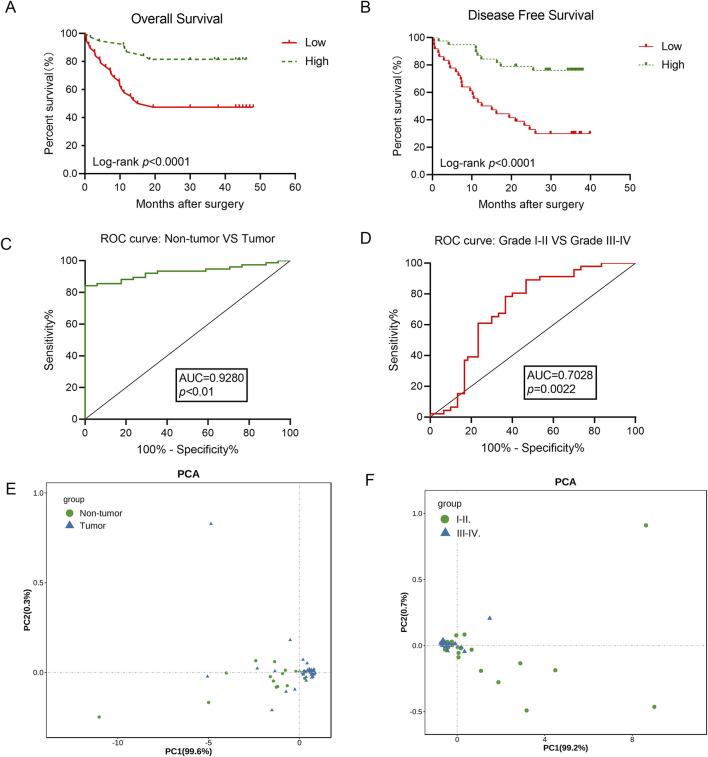
CircMIB1 expression correlates with patient clinical characteristics and has potential as a diagnostic marker for gliomas. **(A)** Overall survival curve, based on the circMIB1 levels, plotted with the Kaplan–Meier method and analyzed using the rank test. **(B)** Disease-free survival curve, based on the circMIB1 levels, plotted with the Kaplan–Meier method and analyzed using the rank test. **(C)** ROC curve of glioma tissue and non-tumor tissue. **(D)** ROC curve of grade I-II tumor from grade III-IV tumor. **(E)** PCA plot consisting of the circMIB1 levels in glioma tissue and non-tumor tissue. **(F)** PCA plot consisting of the circMIB1 levels in grade I–II tumor from grade III–IV tumor. Data are presented as the mean ± SD. *p < 0.05; **p < 0.01; ***p < 0.001; ****p < 0.0001.

To investigate whether circMIB1 levels were associated with clinical features of gliomas, patients with glioma were grouped according to age, sex, and clinical stage; and 76 patients with gliomas were divided into two groups based on the median circMIB1 levels. We found that circMIB1 expression was not statistically correlated with the age and sex of patients with glioma and was strongly associated with the clinical stage (*p* < 0.005). Higher clinical stages were linked to lower expression levels of hsa_circ_circMIB1 (Table 1). According to the log-rank test statistic for survival curves, the survival rate of 76 glioma patients with high expression of circMIB1 was 81.6%, whereas the survival rate of patients with low expression was 47.4%. Thus, the survival rate of glioma patients with high circMIB1 expression was significantly longer than that of patients with low circMIB1 expression. This observation suggested a positive correlation between circMIB1 expression and the overall survival time of patients with glioma (95% confidence interval: 0.6326–0.9543, *p* < 0.0001) (Figure 3A). Similarly, the Kaplan–Meier curve analysis showed that patients with low circMIB1 expression were associated with a higher recurrence rate and shorter time to recurrence (95% confidence interval: 0.8213–0.9511, *p* < 0.0001) (Figure 3B). The same receiver operating characteristic (ROC) curve without adjustment (e.g., age, gender, or tumour type) for other factors showed that circMIB1 could differentiate between non-glioma patients and glioma patients with an area under the curve (AUC) of 0.928 (95% confidence interval:0.855-0.9871; *p* < 0.0001) (Figure 3C). Similarly, CircMIB1 has a relatively low ability to distinguish between LGG patients and HGG patients, with AUC of 0.7028 (95% confidence interval: 0.5571–0.8395; *p* < 0.01) (Figure 3D). Principal component analysis of circMIB1 expression levels was performed to validate the above results, and the results showed that circMIB1 expression levels could effectively differentiate between non-glioma patients and those with glioma (Figure 3E), as well as between patients with LGG and those with HGG (Figure 3F). These results indicate that circMIB1 has potential value as a biomarker for glioma, which needs to be further investigated.

**TABLE 1:** Correlation of circMIB1 expression levels with clinicopathological parameters in glioma patients.

Characteristic	Case	CircMIB1 expression		χ2	*p*-Value
		High	low		
All cases	76	38	38		
Age (years)				0.210	0.646
>50	37	16	21		
≤50	39	22	17		
Gender				1.317	0.251
Male	38	18	20		
Female	38	20	18		
Grade				7.930	**0.005**
I-II	30	21	9		
III-IV	46	17	29		

### Downstream miRNA prediction of circMIB1 in glioma

**FIGURE 4:**
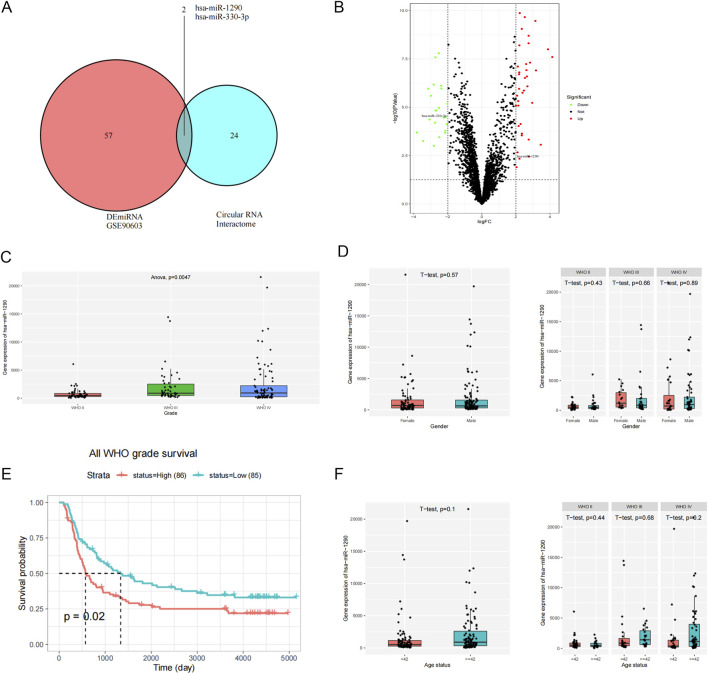
Downstream miRNA prediction of circMIB1 in glioma. **(A)** Predicted target miRNA of circMIB1 based on data obtained from the Circular RNA Interactome and GEO databases. **(B)** Volcano plot of differentially expressed miRNAs predicted to interact with circMIB1. **(C)** Differential analysis of miR-1290 expression in different grades of gliomas. **(D)** Analysis of miR-1290 expression differences between glioma patients of different genders. **(E)** Total survival curve of miR-1290 level. **(F)** Analysis of miR-1290 expression differences between glioma patients of different ages.

CircMIB1 may be involved in the development of glioma through miRNA sponging, thus preventing the inhibitory effect of miRNA on its target genes. To explore the downstream target genes of circMIB1, we first analyzed differentially expressed miRNAs in glioma using GSE90603 RNA-sequencing data from the GEO database (https://www.ncbi.nlm.nih.gov/gds/), and identified 59 differentially expressed miRNA (*p* < 0.05, |log2FC| ≥2) using R language analysis. Subsequently, we predicted downstream miRNAs of circMIB1 by searching a bioinformatics database (Circular RNA Interactome), and performed Venn analysis of these target miRNAs with differential miRNAs in the GEO database. This led to the identification of two miRNAs (hsa-miR-1290 and hsa-miR-330-5p) (Figure 4A). Notably, hsa-miR-1290 was upregulated, whereas hsa-miR-330-5p was downregulated. Of note, hsa-miR-330 inhibits the growth of gliomas, this is not consistent with the normal logic of regulation. Consequently, hsa-miR-1290 was selected for subsequent analysis in this study (Figure 4B). Differential analysis and survival analysis using CGGA (http://www.cgga.org.cn/) prediction combined with miRNAs. Differential and survival analyses were conducted using the CGGA (http://www.cgga.org.cn/) prediction combined with miRNAs. The results showed that hsa-miR-1290 exhibited significant differential expression in different grades of glioma, and the expression levels of hsa-miR-1290 increased with the grade of tumor (*p* < 0.01) (Figure 4C). Nonetheless, there was no significant difference in expression in patients depending on sex and age (Figures 4D, F). Kaplan–Meier curve analysis revealed that the survival of patients with glioma was significantly lower in those with high hsa-miR-1290 expression *versus* those with low hsa-miR-1290 expression. These findings suggested a negative correlation between hsa-miR-1290 levels and the overall survival time of patients with glioma (*p* < 0.05) (Figure 4E).

### CircMIB1 target genes and their expression and survival prognostic value

**FIGURE 5:**
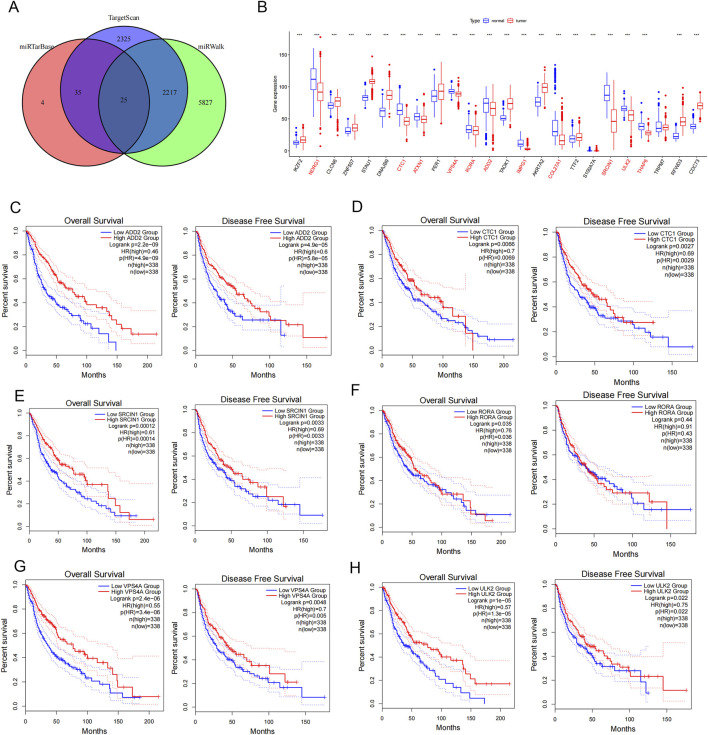
Exploring CircMIB1 downstream genes and their expression and survival prognosis analysis. **(A)** Predicted target mRNA of miR-1290 using data obtained from the miRTarBasesearch, miRWalk, and TargetScan 8.0 databases. **(B)** Differential mRNA expression in glioma tissue and normal brain tissue. **(C)** Overall survival and disease-free survival in ADD2. **(D)** Overall survival and disease-free survival in CTC1. **(E)** Overall survival and disease-free survival in SRCIN1. **(F)** Overall survival and disease-free survival in RORA. **(G)** Overall survival and disease-free survival in VPS4A. **(H)** Overall survival and disease-free survival in ULK2.

We further investigated the potential prognostic value of the ceRNA network in glioma, through miRTarBasesearch (https://mirtarbase.cuhk.edu.cn/), TargetScan8.0 (https://www.targetscan.org/), and the miRWalk (http://mirwalk.umm.uni-heidelberg.de/) to predict the target genes of miR-1290. A total of 25 differentially expressed target genes were identified by the Venn analysis (Figure 5A). We initially analyzed the expression levels of these target genes in normal and tumor tissues using the R “limma” package, which showed that 11 genes were downregulated in glioma tissues (Figure 5B). We further performed survival analysis and disease-free status analysis of differentially expressed genes in glioma using the GEPIA2 to explore the prognostic value of these target genes. The findings revealed that patients with glioma with high expression of adducin 2 (ADD2), CST telomere replication complex component 1 (CTC1), SRC kinase signaling inhibitor 1 (SRCIN1), RAR related orphan receptor A (RORA), vacuolar protein sorting four homolog A (VPS4A), and unc-51 like autophagy activating kinase 2 (ULK2) had a longer survival than those with low expression (Figures 5C–H). Moreover, high expression of ataxin 1 (ATXN1), collagen type XXVII alpha one chain (COL27A1), interphotoreceptor matrix proteoglycan 1 (IMPG1), N-myc downstream regulated 1 (NDRG1), and THAP domain containing 6 (THAP6) offered some advantages in terms of survival (Supplementary Figure S1). Similarly, disease-free survival was found to be longer in patients with glioma with high expression of ADD2, CTC1, SRCIN1, RORA, VPS4A, and ULK2 than in those with low expression (Figures 5C–H). These results suggest that downstream target genes that bind to miR-1290 are associated with survival and prognosis in glioma patients.

### Functional analysis of six downstream target genes of circMIB1

**FIGURE 6:**
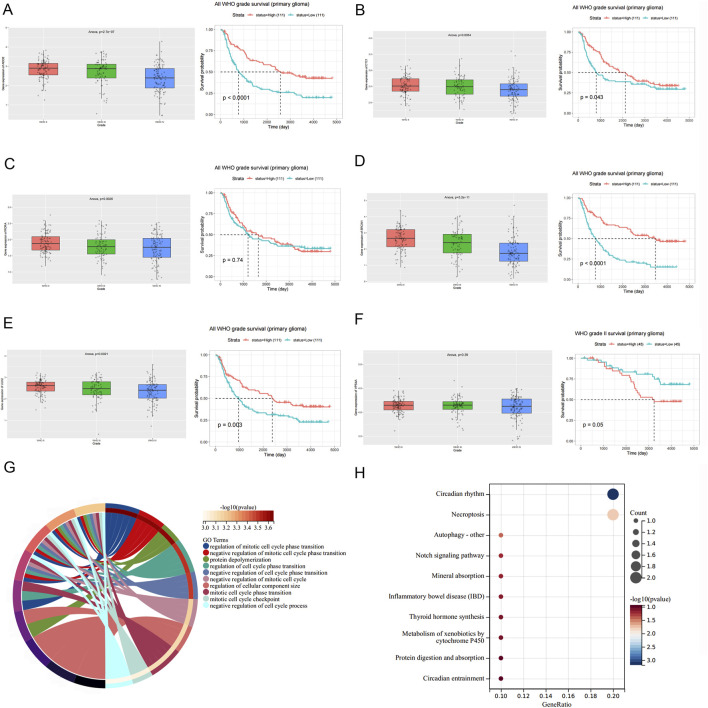
Functional analysis of six downstream target genes of circMIB1. **(A)** Expression and survival analysis of CDD2 in glioma tissues of different grades. **(B)** Expression and survival analysis of CTC1 in glioma tissues of different grades. **(C)** Expression and survival analysis of RORA in glioma tissues of different grades. **(D)** Expression and survival analysis of SRCIN1 in glioma tissues of different grades. **(E)** Expression and survival analysis of ULK2 in glioma tissues of different grades. **(F)** Expression and survival analysis of VPS4A in glioma tissues of different grades. **(G)** GO functional analysis of six downstream target genes. **(H)** KEGG functional analysis of six downstream target genes.

To further investigate the function of circMIB1 downstream target genes, we used the CGGA (http://www.cgga.org.cn/) to validate the expression and prognostic analyses associated with six such genes. We found significant differences in the expression levels of ADD2, CTC1, SRCIN1, RORA, and ULK2 genes in gliomas of different grades, excluding VPS4A (Figures 6A–F). In addition, these genes were generally expressed at higher levels in isocitrate dehydrogenase-mutant (IDH-mutant) gliomas relative to IDH-wild-type gliomas, and were also generally expressed at higher levels in gliomas with chromosome 1p19q deletion than in gliomas without chromosome 1p19q deletion. This evidence implied that the expression of these genes correlates with both the grade of glioma, glioma IDH mutation, and chromosome 1p19q deletion (Supplementary Figure S2).

Furthermore, survival analysis revealed that patients with glioma with high expression of ADD2, CTC1, SRCIN1, VPS4A, ULK2, excluding RORA, had a longer overall survival than those with low expression (Figures 6A–F). Subsequently, we performed GO and Kyoto Encyclopedia of Genes and Genomes (KEGG) enrichment analyses of these genes. Based on the GO enrichment analysis of the dataset, the functions of these genes were mainly related to the cell cycle, and were highly correlated with glioma (Figure 6G). In addition, the KEGG analysis showed that these genes were mainly closely related to the circadian rhythm and apoptotic necrosis (Figure 6H). This provided an alternative explanation for the mechanism underlying the inhibition of glioma by circMIB1. The above results indicate that circMIB1 is involved in the progression of glioma by potentially targeting downstream mRNAs through miR-1290. These data provide suggestions for further investigation of the biological mechanism of circMIB1 involved in the development of glioma.

## Discussion

Gliomas are the most common malignant brain tumors, pathologically characterized by a high degree of morphological and genetic complexity and heterogeneity (Chen et al., 2017). Patients with glioma are linked to a high rate of recurrence, a low survival rate, and a dismal prognosis (Xu et al., 2020; Tom et al., 2019). Therefore, in-depth study of the pathogenesis of glioma and identification of biomarkers and new therapeutic targets are necessary. In recent years, it has been found that circRNA is significantly differentially expressed in glioma (Lu et al., 2019; Sun et al., 2020). Such molecules affect tumor invasion and metastasis, as well as staging and typing, and plays a crucial role in the development of glioma (Liu et al., 2022). In this study, we found that circMIB1 is lowly expressed in glioma tissues and cells, and demonstrated that it correlates with survival prognosis of patients with glioma and has some potential as a biomarker. Moreover, we revealed the molecular mechanism underlying the regulation of the ceRNA network by circMIB1. Furthermore, we emphasized that circMIB1 is closely related to the cell cycle, cellular necrosis, and circadian rhythms, and that it may be a target for the treatment of glioma.

Accumulating evidence suggests that the development of glioma is closely related to the differential expression of circRNAs. Yuan et al. found that 2,038 circRNAs were abnormally expressed between GBM and matched normal brain tissue (Yuan et al., 2018). We similarly found 1,248 differentially expressed circRNA molecules in glioma tissues by high-throughput sequencing, with >87% (n = 1,090) of the circRNAs significantly downregulated. In addition, it was shown that the majority of circRNAs were negatively correlated with tumor proliferation and more opportunistically involved in glioma progression (Bachmayr-Heyda et al., 2015). Therefore, this study focused on circRNAs with downregulated expression. Next, we observed that circMIB1 expression was downregulated and most significantly differentially expressed in glioma cells and tissues by RNA sequencing. CircMIB1 is an emerging molecule that has not been studied and reported in gliomas and other diseases, but its parental protein, MIB1, is an E3 ubiquitin ligase required for the activation of the Notch pathway, and dysregulation of Notch signalling has been associated with cell transformation and tumourigenesis in various types of cancer (Li et al., 2018). Moreover, it has been demonstrated that MIB1 plays an important role in the development of a variety of tumors. For example, in lung cancer, MIB1 is involved in epithelial–mesenchymal transition, cell migration and cell survival (Wang et al., 2022), MIB1 aslo can be used as a marker for the proliferation and prognosis of LGG in children (Gorodezki et al., 2024). Therefore, we predicted that circMBI1 might be involved in the development of gliomas, which was subsequently validated by expanding the sample size by RT-qPCR, and found that circMBI1 expression levels in glioma tissues were significantly downregulated compared with those in normal brain tissues. Therefore, we conjecture that it is important to investigate the mechanism of circMIB1 involvement in glioma development and to determine its role in disease progression and prognosis.

The potential influence of circRNAs on the pathological grading and prognosis of glioma has attracted extensive attention in cancer biology (Sun et al., 2020). The expression levels of hsa_circ_0001649, Circ-ITCH and circNALCN are correlated with the WHO pathological grading of gliomas, and may be an independent prognostic indicator for postoperative patients with glioma (Wang et al., 2018; Liu et al., 2021; Chen et al., 2021). Similarly, our findings showed that circMIB1 was not statistically correlated with age and gender of glioma patients, but was strongly associated with the clinical stage of patients. Notably, these molecules, including circMIB1, will likely be able to assist in diagnosing the clinical staging of gliomas in the future. Additionally, Kaplan-Meier curve analysis, without considering the influence of other factors, showed that glioma patients with high circMIB1 expression had significantly longer survival and lower recurrence rates than those with low expression. These results suggest that circMIB1 expression reflects the degree of malignancy of the tumour and correlates with the survival and prognosis of glioma patients. Hence, this evidence may provide a basis for the clinical treatment of glioma. Recent studies have increasingly focused on the use of circRNAs as cancer diagnostic biomarkers due to their high tolerance to ribonuclease R and high cellular stability (Zou et al., 2020), Zhou et al. found that hsa_circ_ 0004214 has diagnostic value and may be a promising biomarker for glioma (Zhou et al., 2023). We further verified that circMIB1 may be a suitable molecule to be used as a diagnostic biomarker for glioma based on the results of ROC curves and principal component analysis, but the magnitude of its potential remains to be investigated by us further.

Interestingly, circRNAs function as mRNA ‘sponges’, and negatively regulate miRNAs by absorbing and specifically binding to them, thereby increasing the expression of miRNA-associated target genes (Wang et al., 2020; Liu et al., 2022). A study suggested that overexpression of circHIPK3 promotes the proliferation and invasive ability of glioma cells through sponge miR- 124-3p upregulation of signal transducer and activator of STAT3 levels (Hu and Zhang, 2019). Another study also showed that circPTN promotes glioma growth and stemness by sponging miR-145-5p and miR-330-5p, this results in increased expression of NES, CD133, SOX9 and SOX2, which promotes self-renewal of glioma stem cells and regulates gliomagenesis (Chen et al., 2019). Previous studies reported that hsa-miR-1290 is significantly upregulated in glioma cells and tissues and is strongly associated with patient survival and tumor grade (Zhao et al., 2022). Thus, we consider hsa-miR-1290 a downstream regulatory molecule of circMIB1, which in turn inhibits glioma cell growth and affects patient prognosis.

In particular, circRNAs also regulate miRNA sponges and downstream RNA-binding proteins, which participate in and influence the circulatory pathways of glioma progression (Qu et al., 2015). However, the potential function of circRNAs as a ceRNA network and their involvement in glioma warrant further investigation. Hence, we constructed a circRNA-miRNA-mRNA network by bioinformatics analysis, and identified six genes as the central downstream genes in the circMIB1 regulatory axis (i.e., ADD2, CTC1, SRCIN1, VPS4A, ULK2, and RORA). These downstream target genes were differentially expressed in different grades of glioma, exhibited good tumor suppression, and were significantly associated with survival and recurrence rates in patients with glioma. Among these predicted targets, SRCIN1 possesses tumor-suppressive properties in breast cancer and lung cancer inhibiting cell migration and proliferation (Dong et al., 2020; Guo et al., 2019). VPS4A acts as a tumor suppressor by regulating the secretion and uptake of extracellular miRNAs in human hepatocellular carcinoma cells (Wei et al., 2015). ULK1/2 inhibits the actin assembly and hinders breast cancer cell contraction and migration (Liang et al., 2023), Selective splicing of genes in the intronic protein family, including ADD2, leads to alterations in cellular function during pathogenesis are associated with cancer (Kiang and Leung, 2018).

In addition, it has been suggested in the literature that differentially expressed circRNAs are drivers of brain tumors or neurological disorders and are enriched in a variety of cancer-associated signaling pathways (Yuan et al., 2018). Thus, we further explored the functions of the six key genes downstream of circMIB1 and the affected signaling pathways. Results of the GO analysis showed that these six target genes may be associated with the cell cycle, and that disruption of cell cycle regulation is a major mechanism for glioma development and progression (Mason and Cairncross, 2008). Hence, these six genes may be involved in the regulation of the cell cycle, thereby inhibiting tumor development and progression. In addition, KEGG analysis revealed that target genes are mainly involved in pathological processes, such as circadian rhythms and apoptotic necrosis. Studies have found that GBM has a problem with cellular rhythms, and that the circadian gene CSNK1E is a potent target for the treatment of GBM. The growth of GBM can be effectively inhibited by using a CSNK1E inhibitor, which leads to massive death of GBM cells (Varghese et al., 2018). These findings reveal that downstream target genes are closely associated with cancer development and may be involved in different network mechanisms during glioma formation, potentially involving the regulation of circMIB1.

Collectively, our data illustrate that circMIB1 may play an inhibitory role in glioma pathogenesis. Importantly, circMIB1 has the potential to serve as a biomarker for glioma and may play a regulatory role through the ceRNA network. Nevertheless, this study had some limitations. Firstly, the sample size of our cohort study was relatively small and not comprehensive, hence, subsequent studies involving larger sample sizes are warranted. Secondly, functional tests of circMIB1 have not yet been performed *in vitro* and *in vivo*, circMIB1 inhibits glioma growth by participating in the cell cycle, apoptosis, or other pathways, and these functional validation studies would make the findings more reliable. Thus, the present findings only provide a direction to further explore the regulatory pathways underlying the effects of circMIB1 on gliomas. Therefore, in-depth study of the role and exact mechanism of circMIB1 in glioma is our research focus in the future.

In conclusion, we demonstrated that circMIB1 is lowly expressed in glioma tissues and cells, and that its expression is closely related to the survival prognosis of patients. Thus, circMIB1 may be able to serve as a target for glioma diagnosis and treatment. In addition, circMIB1 may also regulate the differential expression of downstream hsa-miR-1290 and related target genes, which are further involved in glioma progression. Further understanding of the specific role of circMIB1 in tumour grading and treatment through ceRNA networks will provide new perspectives and approaches for early diagnosis and treatment of gliomas.

## Data Availability

The datasets presented in this study can be found in online repositories. The names of the repository/repositories and accession number(s) can be found in the article/Supplementary Material.
