# Corrigendum: CircMIB1 inhibits glioma development and progression through a competing endogenous RNA interaction network

**DOI:** 10.3389/fmolb.2025.1599157

**Published:** 2025-04-16

**Authors:** Simin Chen, Longping Li, Wei Xu, Nanjiao Xie, Huiting Xu, Yongjun Zhou, Ying Zou, Kai Yi, Zuping Zhang

**Affiliations:** ^1^ Department of Clinical Laboratory, Yiyang Central Hospital, Yiyang, Hunan, China; ^2^ School of Xiangya Basic Medical Science, Central South University, Changsha, Hunan, China

**Keywords:** CircMIB1, glioma, MiR-1290, ceRNA, prognostic, biomarker

In the published article, there was an error in the **Author list**. Corresponding author Zuping Zhang was erroneously excluded and author Yi Liu was erroneously included. The corrected **Author list** appears below. The correspondence section has been updated accordingly.

Simin Chen^1†^, Longping Li^1†^, Wei Xu^1^, Nanjiao Xie^1^, Huiting Xu^1^, Yongjun Zhou^1^, Ying Zou^1^, Kai Yi^1^, Zuping Zhang^2^* 

In the published article, there was an error in **Affiliation 2**. Instead of “Department of Emergency Medicine and Laboratory of Emergency Medicine, West China Hospital, West China School of Medicine, Sichuan University Chengdu, Chengdu, China”, it should be “School of Xiangya Basic Medical Science, Central South University, Changsha, Hunan, China”.

In the published article, there was an error in the **Author contributions**. The updated statement appears below:

**AUTHOR CONTRIBUTIONS**

SC: Funding acquisition, Conceptualization, Data curation, Formal Analysis, Investigation, Methodology, Supervision, Visualization, Writing – original draft, Writing – review and editing. LL: Resources, Formal Analysis, Methodology, Visualization, Writing – original draft, Writing – review and editing. WX: Software, Investigation, Methodology, Writing – original draft. NX: Software, Investigation, Writing – original draft. HX: Software, Investigation, Writing – review and editing. YoZ: Investigation, Writing – review and editing. YiZ: Software, Writing – review and editing. KY: Software, Writing – review and editing. ZZ: Resources, Investigation, Funding acquisition, Supervision, Conceptualization, Writing – review and editing.

In the published article, there was an error in the **Funding**statement. Due to an oversight on the part of the first author, the name of funder does not correspond to the official one. The correct **Funding**statement appears below.

**FUNDING**

The author(s) declare that financial support was received for the research and/or publication of this article. Our research was supported by Hunan Provincial Natural Science Foundation of China (No. 2024JJ9575).

The authors apologize for these errors and state that these do not change the scientific conclusions of the article in any way. The original article has been updated.

