# Supplementary material for: CircMIB1 inhibits glioma development and progression through a competing endogenous RNA interaction network
Source: Front Mol Biosci. 2024 Dec 4;11:1513919. doi: 10.3389/fmolb.2024.1513919 (PMC11652353; doi:10.3389/fmolb.2024.1513919)
Supplement: Supplementary file 1 [file Table1.docx]

**Table S1 Primer sequences**

| Genes | Primer | Sequence (5’-3’) |
| --- | --- | --- |
| circMIB1 (divergence) | Forward primer | ATCCAAGTGGCAATAGGCAT |
|  | Reverse primer | GCACCTGCAAAGATTCCTC |
| circMIB1 (convergence) | Forward primer | CTTTGCAGCATGGTCATGG |
|  | Reverse primer | TCCATCATGCTTGATGCCTA |
| GAPDH (convergence) | Forward primer | GTCTCCTCTGACTTCAACAGCG |
|  | Reverse primer | ACCACCCTGTTGCTGTAGCCAA |
| GAPDH (divergence) | Forward primer | TGTTCTGTTTCGTGTGTGAGG |
|  | Reverse primer | TGTGAGAAGCCTCTGTTCGT |
| MIB1 | Forward primer | ACTGGCAGTGGGAAGATCAA |
|  | Reverse primer | AGAAAGAACCTCCCTTGGCA |
| has_circ_0117874 | Forward primer | GAAGGACCATGCTCAGAAGC |
|  | Reverse primer | ATTAACTCTTCCATTGCAGC |
| has_circ_0114651 | Forward primer | TCACTGCAAAGACCACACAG |
|  | Reverse primer | GGCGTTCCAGTTTCATGCG |
| has_circ_0112695 | Forward primer | GCCATTCAAACTGCTATCCG |
|  | Reverse primer | GTAACAATCCTGACTTGGTAGCT |
| has_circ_0099761 | Forward primer | AGATCTGGGACTTAGCAGGC |
|  | Reverse primer | GCAAGCACAAAACCACAGTCTA |
| has_circ_0092794 | Forward primer | TTTCCTGGCCTATTAAAATACAGATG |
|  | Reverse primer | TTCGACTGTTCTGGGTTTG |
| has_circ_0000835 | Forward primer | ATCCAAGTGGCAATAGGCAT |
|  | Reverse primer | GCACCTGCAAAGATTCCTC |
| has_circ_0007061 | Forward primer | AATGGTGACAGATCCAGGCA |
|  | Reverse primer | GATTCCAGCAGCCTCCTG |
