# Supplementary material for: CircMIB1 inhibits glioma development and progression through a competing endogenous RNA interaction network
Source: Front Mol Biosci. 2024 Dec 4;11:1513919. doi: 10.3389/fmolb.2024.1513919 (PMC11652353; doi:10.3389/fmolb.2024.1513919)
Supplement: Supplementary file 2 [file DataSheet1.pdf]

Supplementary Figures

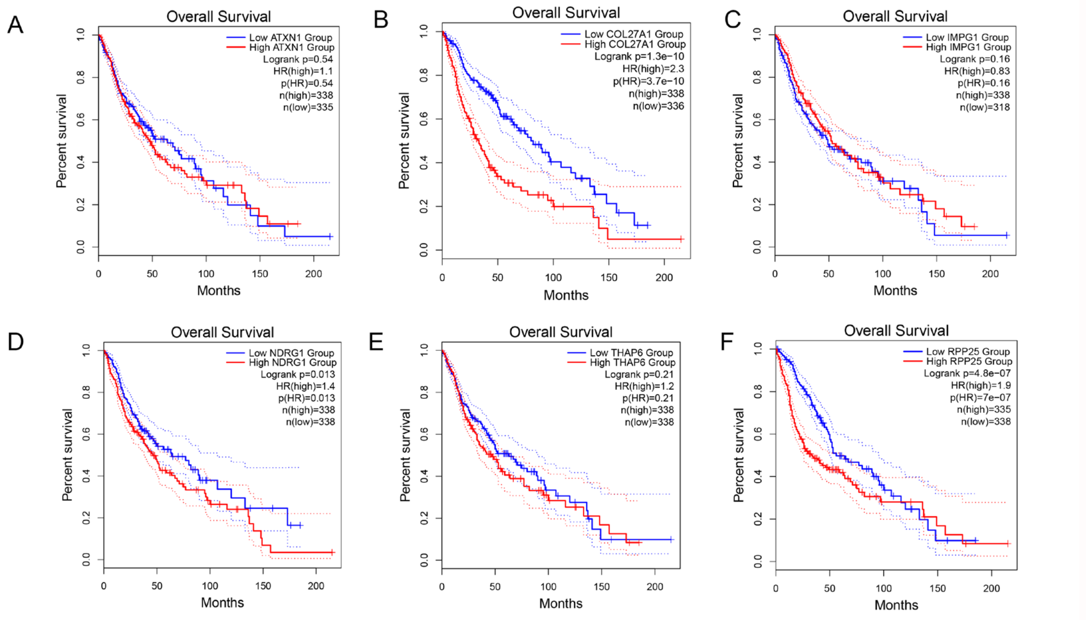

Supplementary Figure 1. Survival analysis of miR-1290 downstream gene in glioma.

(A) Overall survival in ATXN1. (B) Overall survival in COL27A1. (C) Overall survival in IMPG1. (D) Overall survival in NDRG1. (E) Overall survival in THAP6. (F) Overall survival in RPP25.

Supplementary Figure 2. Expression level of six downstream target genes of circMIB1.

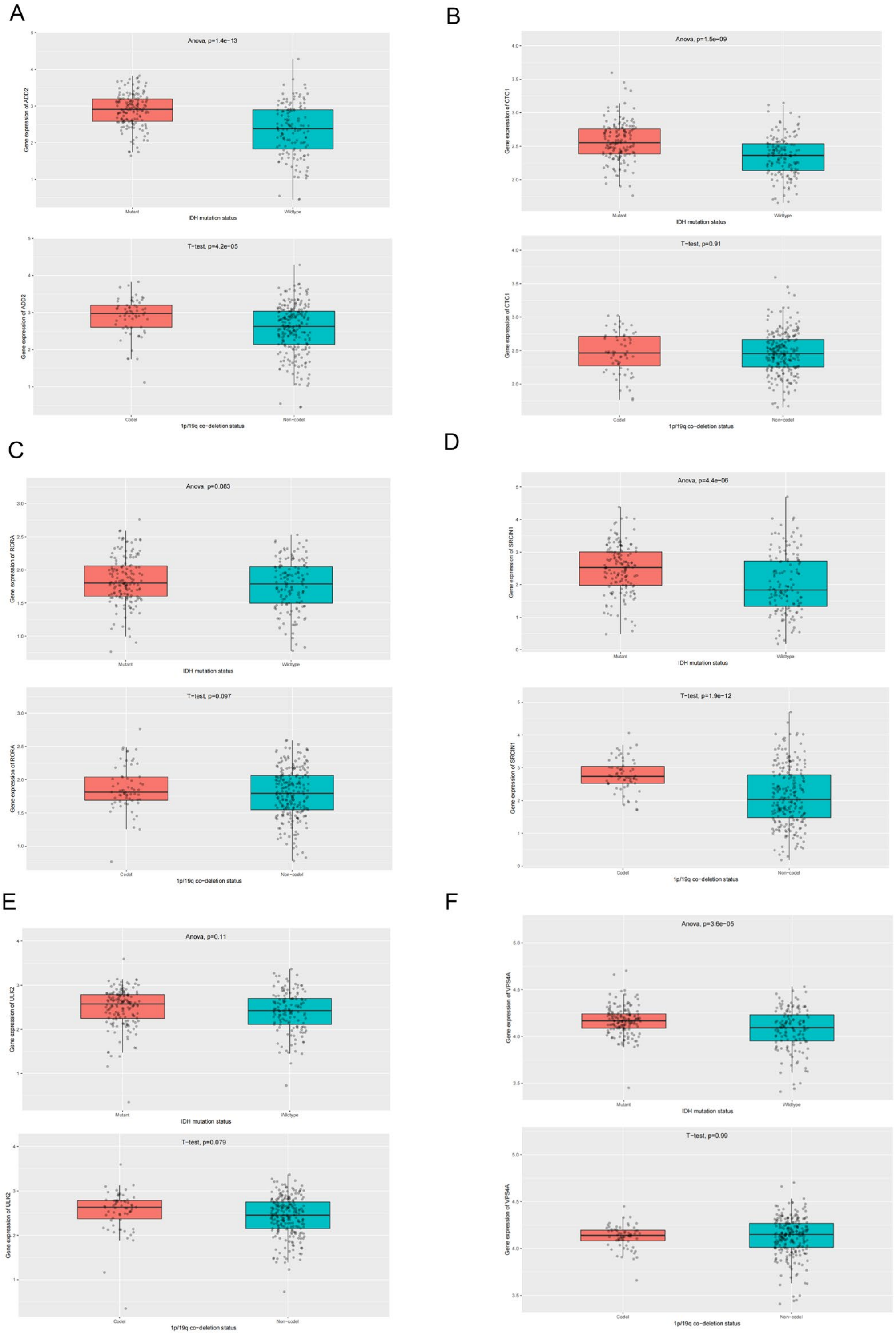

(A) Expression levels of ADD2 in IDH with or without mutation status and chromosome 1p19q with or without deletion status. (B) Expression levels of CTC1 in

IDH with or without mutation status and chromosome 1p19q with or without deletion status. (C) Expression levels of RORA in IDH with or without mutation status and chromosome 1p19q with or without deletion status. (D) Expression levels of SRCIN1 in IDH with or without mutation status and chromosome 1p19q with or without deletion status. (E) Expression levels of ULK2 in IDH with or without mutation status and chromosome 1p19q with or without deletion status. (F) Expression levels of VPS4A in IDH with or without mutation status and chromosome 1p19q with or without deletion status.
